# Prospective Long-Term Cohort Study of Subjects With First-Episode Psychosis Examining Eight Major Outcome Domains and Their Predictors: Study Protocol

**DOI:** 10.3389/fpsyt.2021.643112

**Published:** 2021-03-19

**Authors:** Victor Peralta, Lucía Moreno-Izco, Elena García de Jalón, Ana M. Sánchez-Torres, Lucía Janda, David Peralta, Lourdes Fañanás, Manuel J. Cuesta, SEGPEPs Group

**Affiliations:** ^1^Mental Health Department, Servicio Navarro de Salud, Pamplona, Spain; ^2^Instituto de Investigación Sanitaria de Navarra (IdiSNA), Pamplona, Spain; ^3^Department of Psychiatry, Complejo Hospitalario de Navarra, Pamplona, Spain; ^4^Department of Evolutionary Biology, Ecology and Environmental Sciences, Faculty of Biology, University of Barcelona, Barcelona, Spain; ^5^Centro de Investigación Biomédica en Red en Salud Mental (CIBERSAM), Madrid, Spain

**Keywords:** psychosis, risk factors, follow-up, outcome, remission, recovery, predictive model, clinical staging

## Abstract

**Background:** Our current ability to predict the long-term course and outcome of subjects with a first-episode of psychosis (FEP) is limited. To improve our understanding of the long-term outcomes of psychotic disorders and their determinants, we designed a follow-up study using a well-characterized sample of FEP and a multidimensional approach to the outcomes. The main goals were to characterize the long-term outcomes of psychotic disorders from a multidimensional perspective, to address the commonalities and differential characteristics of the outcomes, and to examine the common and specific predictors of each outcome domain. This article describes the rationale, methods, and design of a longitudinal and naturalistic study of subjects with epidemiologically defined first-admission psychosis.

**Methods:** Eligible subjects were recruited from consecutive admissions between January 1990 and December 2009. Between January 2018 and June 2021, we sought to trace, re-contact, and re-interview the subjects to assess the clinical course, trajectories of symptoms and functioning, and the different outcomes of psychotic disorders. Since this is a naturalistic study, the research team will not interfere with the subjects' care and treatment. Predictors include antecedent variables, first-episode characteristics, and illness-related variables over the illness course. We assess eight outcome domains at follow-up: psychopathology, psychosocial functioning, self-rated personal recovery, self-rated quality of life, cognitive performance, neuromotor dysfunction, medical and psychiatric comorbidities, and mortality rate. The range of the follow-up period will be 10–31 years with an estimated mean of 20 years. We estimate that more than 50% of the baseline sample will be assessed at follow-up.

**Discussion:** The study design was driven by the increasing need to refine the ability to predict the different clinical outcomes in FEP, and it aims to close current gaps in knowledge, with a broad approach to both the definition of outcomes and their determinants. To the best of our knowledge, this study is one of the few attempting to characterize the very long-term outcome of FEP and the only study addressing eight major outcome domains. We hope that this study helps to better characterize the long-term outcomes and their determinants, enabling better risk stratification and individually tailored, person-based interventions.

## Introduction

The estimated lifetime prevalence of psychotic disorders is 3.5% (1), and they are among the most severe illnesses in terms of individual, familial, and societal impact (2, 3). Heterogeneity is the rule rather than the exception in psychotic disorders, which is manifested at the levels of risk factors, clinical manifestations, course, and outcome. Furthermore, using data from 11 studies, Hjorthøj et al. (4) showed that schizophrenia was associated with an average of 14.5 years of potential life lost. The outcome of first-episode psychosis (FEP) is highly variable, ranging from full symptomatic remission to a chronic course and substantial psychosocial impairment. For clinicians, the possibility of predicting patient outcomes would be highly valuable for tailoring drug and psychosocial treatments (5). Thus, the challenge for the clinician is how to predict the varied outcomes based on the subject's first-episode characteristics and their background risk factors to predict the outcomes and make the best treatment choices for individual patients.

Current diagnostic systems of psychotic disorders are challenged by issues such as poor diagnostic stability, clinical heterogeneity, non-specific treatment effects, and lack of diagnosis-specific neurobiological markers (6, 7). Indeed, attempting to resolve the heterogeneity question of psychotic disorders with traditional diagnostic categories is like “trying to clarify a cloudy liquid simply by pouring it from one container to another” (8) (p. 542). Furthermore, it is increasingly recognized that psychopathology is expressed dimensionally, that psychotic disorders lack clear nosological borders and that their clinical manifestations evolve gradually in a dynamic way that is influenced by a variety of antecedent and life-course variables (9). A model designed to capture this continuity of both severity and time is the clinical staging model (10). This model describes established psychotic disorders as ranging, through subsequent but qualitatively different stages, from full remission at the lowest level, to progressive stages of severity potentially reaching a chronic and continuous stage. The clinical staging model has been mainly applied to the early stages of psychotic disorders, the so-called critical period (11), but it has barely been applied to the long-term course of psychotic disorders. More recently, new advances in epistatic gene × environment interactions have provided important insights into the complex aetiopathological basis of schizophrenia by the development of polygenic (12) and polyenviromic (13, 14) risk scores, which are of potential utility to predict the long-term outcome of psychotic disorders (15, 16).

Despite epidemiologically robust studies, data on the outcomes of FEP have been limited by the scarcity of very long-term (≥20 years) follow-up designs and the limited measures of outcome utilized. The majority of studies of the long-term outcomes of the psychoses have been limited to symptomatic or psychosocial recovery (17, 18). Only a few studies have evaluated the long-term outcome of psychotic disorders from a multidimensional perspective (19–21), but they rarely examined conjointly the background and baseline predictors of the different outcomes, including genetic factors, familial loading of psychotic disorders, perinatal factors, premorbid dysfunction, proximal risk factors, and first-episode characteristics, including early response to antipsychotic medication. Thus, a comprehensive approach to the early predictors of the long-term outcomes of psychotic disorders is a clear unmet need. Furthermore, contemporary mental health policy and service users are promoting the recovery-oriented approach, which extends beyond symptomatology and functioning to incorporate self-reported personal recovery (22). Several authors emphasized that symptomatic remission, functional remission, and personal recovery are distinct, although to some extent, overlapping concepts (21, 23). However, the degree to which the early predictors of these and other outcome constructs differ remains largely unknown (24–26). Other major outcome areas also include quality of life, cognitive functioning, neuromotor dysfunction, medical and psychiatric comorbidities, and premature mortality. All of these outcomes will be examined in our study and described in the present protocol.

### Aims and Hypotheses

This study was designed to overcome the aforementioned challenges by adopting a transdiagnostic and comprehensive approach to the long-term outcome of psychotic disorders and their determinants using a well-characterized sample of FEP admissions. The main goals of our study were, (a) to characterize the long-term outcome of psychotic disorders from a multidimensional perspective, (b) to address the commonalities and differential characteristics of the outcomes, and (c) to examine the common and specific predictors of each outcome domain. The secondary aims were to examine the stability of specific diagnoses and the determinants of diagnostic change between the diagnoses at the FEP and the diagnoses at follow-up, and to validate a new definition of clinical staging based on the long-term outcome using the background and first-episode variables as the validators.

Based on previous evidence indicating that the different outcomes are independent or semi-independent constructs, we hypothesize that different antecedent, baseline, and mediating variables will predict different outcomes to some extent. It was also hypothesized that different clinical stages at follow-up will be differentially predicted by the validators or by the same validator in a dose-response fashion.

We present here the protocol of an innovative, long-term cohort study of subjects with a first-episode of psychosis (FEP) who are being followed for a mean of 20 years. The study design was driven by an increasing need to refine the ability to predict different clinical outcomes in this population and aims at filling current gaps in knowledge, with a broad approach to both definition of outcome and their determinants.

## Methods

### Study Design and Population

This was a longitudinal and naturalistic study of subjects with epidemiologically defined first-admission psychosis. Eligible subjects were consecutively admitted to Psychiatric Unit B of the Complejo Hospitalario of Navarra in Pamplona (Spain) between January 1990 and December 2008. The hospital serves a catchment area for approximately 200,000 inhabitants of a predominantly urban region.

Between January 2018 and June 2021 we sought to trace, re-contact, and re-interview the subjects to assess the clinical course, trajectories of symptoms and functioning, and different outcomes of the psychotic Illness. Since this is a naturalistic study, the research team will not interfere with the subjects' care and treatment. All the subjects assessed at follow-up were treated according to clinical choice over their illness course.

### Eligible Subjects at Baseline

The baseline study cohort was made of subjects fulfilling the following inclusion criteria: (a) being admitted for a FEP fulfilling DSM-III-R or DSM-IV criteria for a functional psychotic disorder; (b) age 15–65 years old; (c) residing in the catchment area of the hospital; (d) completing the inpatient treatment period and a 6-month assessment after discharge to re-evaluate diagnosis, symptom status, and functioning; (e) close relatives available to provide broad background information; and (f) written informed consent. The exclusion criteria comprised: (a) previous antipsychotic treatment for more than 2 months; (b) drug-induced psychotic disorder; (c) history of serious medical or neurological disease, including head injury with loss of consciousness; and (d) mental disability as defined by an IQ <70.

We initially interviewed 623 subjects who were admitted for a FEP during the recruitment period, 510 of them met the eligibility criteria and made the baseline study sample. Excluded subjects (*n* = 113) did not significantly differ from the eligible subjects in terms of age, gender, and DSM diagnosis of psychotic disorder. For all the eligible subjects, this admission was the first and 393 (77.1%) were antipsychotic drug-naïve at admission to the hospital. The DSM-5 diagnoses of the baseline sample was as follows: schizophrenia (*n* = 161), schizophreniform disorder (*n* = 79), brief psychotic disorder (*n* = 81), delusional disorder (*n* = 39), schizoaffective disorder (*n* = 25), mania/bipolar disorder with psychotic features (*n* = 43), major depression with psychotic features (*n* = 59), and psychotic disorder not otherwise specified (*n* = 23).

### Tracing and Re-Contact Procedures for the Follow-Up

We began by identifying the subjects currently in contact with mental health services across the whole region of Navarra. We next sent letters to their last known address, inviting them to participate. Non-responders were contacted by telephone if the number was available in the health records. Non-responders to the first letter or to the telephone call were sent a further letter 2 months later. Finally, for those identified cases who did not respond, we sought to make contact and invite them via their treating psychiatrist or general practitioner. All deaths were identified via electronic health records or the General Register Office. If the subject expressed an interest in the study, he or she were invited by phone to meet the field raters to learn about and discuss participation. Through November 30, 2020, we have re-assessed 216 subjects, whereby we expect to recruit more than 50% of the subjects from the baseline sample, a figure in line with other long-term follow-up studies (21).

### Assessment Methodology and Raters

At baseline, all the subjects were clinically assessed by the senior authors (VP or MJC), who were also their treating psychiatrists during the inpatient treatment period in 87% of the cases.

The follow-up field interviewers (LMI and EGJ) were clinical psychiatrists with more than 15 years of clinical practice and experience in assessing psychotic subjects with standardized rating scales, including the Comprehensive Assessment of Symptoms and History (CASH) (27, 28), which was the main assessment instrument across all study stages. Field interviewers were blind to the first-episode characteristics of each subject and their background information. They conducted face-to-face interviews with each subject, consulted clinical records, and interview significant others. This multisource information was utilized to rate the clinical status of the subjects at follow-up and to characterize outcomes.

To delineate the clinical course, psychopathology and functioning over the illness course (from 6 months after the index admission to the follow-up interview), the primary measure instruments were the Past and Lifetime History sections from the CASH, which were used to construct a Life Chart Schedule (LCS) for each subject. These CASH sections are based on the WHO-LCS (20), which standardizes retrospective assessment with high reliability (29) and allows for constructing lifetime trajectories of symptoms, functioning and other illness-related variables, such as medication history, medical and psychiatric comorbidities, drug abuse, major life events, and service use over the entire illness course. The LCS documented the data after the first admission using a year-by-year follow-back procedure, summarized into five time-periods: the first 2 years after index admission, years 3–5, years 6–10, years 11–15, and years 16–20. More specifically, we assessed symptomatic and functional remission status at month 6 and years 2, 5, 10, 15, and 20, considering the last 6 months to assess symptom remission according to the Remission in Schizophrenia Working Group (RSWG) criteria (30, 31). Functional remission was assessed using the Global Assessment of Functioning (GAF) scale (32), and a score >60 was indicative of functional remission.

The life chart raters were six psychiatrists with at least 5 years of clinical practice. They collected information in interviews with the subjects, key informants, extensive clinical records and, when appropriate, the treating physician. Clinical records were reviewed through the computerized database of the local health service, which covers all public medical and mental health services in Navarra. Raters were blind to baseline and outcome measures and used clinical judgment to assess discrepancies in information. When the subject was unable to recall major distal events, the main sources of information were the clinical records and the key informants.

Raters completing life charts and follow-up assessments were specifically trained by the senior authors in administering the CASH and other rating scales at regular training sessions before starting the follow-up visit. Adequate interrater reliability for the CASH ratings has been demonstrated in our center (33). However, rather than examining inter-rater reliability among all study's raters, we opted to discuss cases at regular meetings to minimize information and criterion variance. The researchers presented detailed summaries of the subjects, discrepancies, or uncertainties were discussed, and consensus ratings were reached. Final lifetime diagnoses were made by consensus between the two senior authors using all available information.

### Baseline Assessments

Rating instruments by time period assessed are summarized in the Table 1. The main instrument for assessing baseline sociodemographic variables, antecedents, premorbid adjustment, symptoms, and diagnosis was the CASH (27, 28). The CASH is a structured interview and recording instrument for documenting a broad range of illness-related factors of subjects with psychotic and major mood disorders. Its major emphasis is to provide broad descriptive coverage to make diagnosis using a variety of criteria, which is especially important because of the changing diagnostic systems. In this manner, we could diagnose all the subjects at baseline using the DSM-III-R (34) or DSM-IV (35) criteria and re-diagnose them with the DSM-5 (36) criteria using all information contained within the CASH.

**Table 1:** Overview of assessment instruments by time-periods.

	**Instrument**	**Purpose**	**Basal**	**6-Month**	**Year 2**	**Year 5**	**Year 10**	**Year 15**	**Year 20**	**Follow-up**
Demographics	CASH	Confounders (age, gender, education)	X							X
Familial-genetic factors	PRS	SZ, BIP, Cognition								X
	FLS	Familial load of SZ, BIP and MDD	X							X
Distal antecedents	L-M	Obstetric complications	X							
	Register	Season of birth	X							
	NDS	Neurodevelopmental delay	X							
Intermediate antecedents	CASH	Childhood adjustment	X							
	GFES	Childhood adversity	X							
	C-SPAS	Scholastic performance	X							
	CASH	Adolescence adjustment	X							
	CASH	Deterioration in adjustment	X							
	GAF	Premorbid global functioning								
Proximal antecedents	DSM-III	Acute psychosocial stressors	X							
	CASH, ASS	Type and severity of drug abuse	X							
Illness-onset factors	CASH	Duration of untreated psychosis	X							
	CASH	Mode of onset	X							
	CASH	Age at illness onset	X							
Treatment response	CGI-EI, RSWG	Early treatment response at discharge	X							
	RSWG	6-Month treatment response		X	X	X	X	X	X	X
Illness-course variables	CASH, DSM-5	Diagnostic class of psychotic disorder	X	X	X	X	X	X	X	X
	CASH,	Treatment history	X	X	X	X	X	X	X	X
	CASH, ASS	Drug abuse	X	X	X	X	X	X	X	X
	CASH	Service use	X	X	X	X	X	X	X	X
	CASH	No. and timing of relapses		X	X	X	X	X	X	X
	CASH	No. of suicide attempts	X	X	X	X	X	X	X	X
	CASH	Typification of illness course	X	X	X	X	X	X	X	X
	CASH	Lifetime symptom load			X	X	X	X	X	X
Lifetime comorbidity	Register	Other mental disorders			X	X	X	X	X	X
	Register	Medical illnesses and treatments								X
Psychopathology	CASH	Psychotic and mood symptoms	X	X	X	X	X	X	X	X
	PANSS	Lack of insight	X							
	SDS	Deficit symptoms			X	X	X	X	X	X
	BADDS	Lifetime mood and psychotic symptoms								X
Motor dysfunction	SARS	Parkinsonism	X							X
	AIMS	Dyskinesia	X							X
	BARS	Akathisia	X							
	MRS	Motor abnormalities	X							X
	NES	Neurological soft signs	X							X
Psychosocial functioning	GAF	Global symptoms and functioning	X	X	X	X	X	X	X	X
	SOFAS	Social and occupational functioning			X	X	X	X	X	X
	WHO-DAS	Four areas of functioning			X	X	X	X	X	X
	FAST	Six areas of functioning	X		X	X	X	X	X	X
Symptomatic remission	CASH	RSWG criteria		X	X	X	X	X	X	X
Functional remission	GAF	GAF score ≥61		X	X	X	X	X	X	X
Personal recovery	PRQ-15	Self-reported recovery								X
Quality of life	SQLS	Self-reported quality of life								X
	EUROQOL	Self-reported health dimensions								X
Cognition	SCIP	Brief cognitive screening								X
	MoCA	Brief neuro-cognitive screening								X
	MCCB	Extensive cognitive battery								X
Metabolic syndrome	AHA criteria	Cardio-metabolic risk factors								X

*AHA: American Heart Association; AIMS: Abnormal Involuntary Movements Scale; ASS: Addition Severity Scale BADDS: Bipolar Dimension Disorder Scale; BARS: Barnes Akathisia Rating Scale; BIP: Bipolar Disorder; CASH: Comprehensive Assessment of Symptoms and History; CGI-EI: Clinical Global Impression Efficacy Index; C-SPAS: Cannon-Spoor Premorbid Adjustment Scale; EUROQOL: European Quality of Life Group; FAST: Functioning Assessment Short Test; FLS: Familial Load Score; GAF: Global Assessment of Functioning; GFES: Global Family Environment Scale; L-M: Lewis-Murray Obstetric Complications Scale; MDD: Major Depressive Disorder; MoCA: Montreal Cognitive Assessment; MRS: Modified Rogers Scale; NDS: Neurodevelopmental Scale; NES: Neurological Evaluation Scale; PANSS: Positive and Negative Syndrome Scale; PRQ-15: Questionnaire about the Process of Recovery; PRS: Polygenic Risk Score; RSWG: Remission in Schizophrenia Working Group; SARS: Simpson-Angus Rating Scale; SDS: Schedule for the Deficit Syndrome; SOFAS: Social and Occupational Functioning Assessment Scale; SQLS: Schizophrenia Quality of Life; SZ: Schizophrenia; WHO-DAS: World Health Organization-Disability Assessment Schedule. Assessments at year 2, 5, 10, 15 and 20 were made using a retrospective life charting methodology*.

An important feature of the CASH is the broad symptom coverage, including 73 symptoms rated at their worst over the previous month on a 6-point scale. It includes the Scale for the Assessment of Positive Symptoms (SAPS), the Scale for the Assessment of Negative Symptoms (SANS), five catatonic signs and a global severity rating for the syndrome, ten depressive symptoms and a global severity rating for the syndrome, and eight manic symptoms and a global rating for the syndrome. Thus, the current condition examination from the CASH has a hierarchical structure in that the item-level symptoms are summarized into global ratings, which in turn can be summarized as syndromes. For instance, the 12 items describing delusions are summarized into a single global severity rating for delusions, which together with the global rating for hallucinations delineate the reality-distortion syndrome. Altogether, six syndromic global ratings can be distinguished within the CASH: reality-distortion, disorganization, negative, catatonia, mania, and depression.

**Figure 1:**
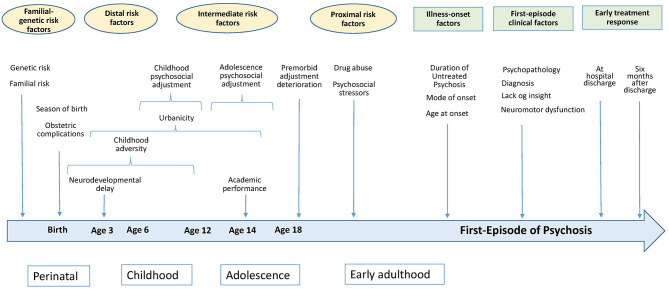
A schematic time-line representation of baseline predictors of long-term outcomes of psychotic disorders.

Acknowledging that there is a continuum of the timing of antecedents and illness-onset factors, antecedent variables were subdivided according to their distance/proximity to the psychotic breakdown into familial-genetic factors, distal antecedents, intermediate antecedents, and proximal antecedents. In turn, first-episode variables were subdivided into illness-onset factors, clinical state, and response to treatment (Figure 1).

#### Familial-Genetic Factors

Polygenic risk scores (PRSs) will be calculated for schizophrenia, bipolar disorder, and cognition using DNA samples obtained at follow-up, which will be genotyped using the Global Screening Array from Illumina.

We estimate the familial load score for schizophrenia, bipolar disorder, and major depression in the first-degree relatives of the subjects, which is calculated by means of the Family History-Research Diagnostic Criteria (37) considering family size and age structure (38). Family history is assessed at baseline and follow-up, and the two assessments were collated to compute the familial load scores.

#### Distal Antecedents

Obstetric complications were rated with the Lewis and Murray scale (39). The assessment of developmental milestones attainment at age 3 was based on Shapiro et al. (40) and included ratings for six specific milestones: sitting, standing, walking, talking words, talking sentences, and urine/faces control. We derived a Neurodevelopmental Delay Score (NDS) ranging from 0 (all milestones attained at the expected age) to 6 (none of the milestones attained at the expected age). Obstetric complications and neurodevelopmental attainment were rated using information from clinical registers and the subject's mother, who was available in the majority of the cases.

#### Intermediate Antecedents

These antecedents comprise different premorbid events and functioning up to age 18. The modified Gittelman-Klein scale (as included in the CASH) was used to rate premorbid social adjustment during childhood (ages 6–12) and adolescence (ages 13–18). We also calculated deterioration in premorbid functioning at age 18 by subtracting the childhood score from the adolescence score.

Scholastic performance is rated at age 14 using the corresponding subscale from the premorbid adjustment scale (41), scored from 1 (excellent student) to 7 (failing all classes). Childhood adversity was assessed by means of the Global Family Environment Scale (GFES) (42), which rates the quality of the rearing environment of the child until age 12. The GFES was not available at the beginning of the recruitment period; thus, in 28% of the cases ratings were made using the rich available background information on this variable.

Urbanicity during upbringing until age 15 is scored from 1 (rural area, <5,000 inhabitants) to 3 (urban area, >100,000 inhabitants).

#### Proximal Antecedents

Proximal antecedents are conceptualized as trigger factors that occur within the 6 months before illness onset. They include acute psychosocial stressors rated per DSM-III Axis IV (43), which is scored from 1 (absent) to 7 (catastrophic), and substance abuse or dependence as rated per CASH. Severity of drug abuse for alcohol, cannabis, cocaine, stimulants, heroine, hallucinogens, other drugs and a global severity rating are also scored using the Addiction Severity Scale (44). We constructed an environmental risk score on the basis of season of birth, obstetric complications, urbanicity level, childhood adversity, drug abuse, and psychosocial stressors scores.

#### Illness-Onset Factors

Age at illness onset was defined as the age at which the subject met DSM criterion A for schizophrenia. Duration of untreated psychosis (DUP) was defined as the months that elapsed between the first psychotic symptom and the first adequate antipsychotic treatment. Duration of untreated continuous psychosis (DUCP) was defined as the months that elapsed between the first continuous psychotic symptom, defined as present most of the time, and the first adequate antipsychotic treatment Mode of onset was rated from 1 (acute, <1 month) to 4 (>6 months), indicating the time between the onset of any illness-related symptom and the age at illness onset.

To monitor more closely the subjects' deterioration in functioning close to the FEP, we used the overall functioning scale from the CASH rated 1 (good functioning in all areas) to 6 (major impairment in several areas) at 1, 3, and 5 years before the index admission.

#### First-Episode Clinical State

The type of admission (voluntary or involuntary) was registered, and the level of awareness of illness at index admission was evaluated using the item “lack of insight” from the Positive and Negative Symptom Scale (45).

Psychopathological assessment consisted of a cross-sectional 3-point measurement of symptoms with the CASH, which was administered at admission, discharge, and 6 months after discharge. For coherence with further assessments, psychopathology is summarized using the well-established syndrome domains of reality-distortion, disorganization, negative, catatonia, mania, and depression, each rated on a 0 (absent) to 5 (severe) scale.

The same rating scales to assess neuromotor dysfunction at follow-up (see below) were also used at index admission (Table 1). In the subjects who were drug-naïve at index admission, and before incepting antipsychotic medication, the so-called spontaneous or primary motor abnormalities, including dyskinesia and Parkinsonism, were rated.

#### First-Episode Response to Treatment

We distinguished between early and 6-month response to treatment. At first-admission subjects were treated according to clinical choice, and early treatment response was rated at discharge using both the RSWG criteria without considering the 6-mont criterion, and the Clinical Global Impression Efficacy Index (CGI-EI) (46), which rates the degree of symptomatic improvement from 1 (marked remission) to 4 (unchanged). At 6 months after discharge, response to treatment was rated according the RSWG criteria.

### Life Chart Schedule Assessment

#### Trajectories of Symptoms and Functioning

Symptom and functioning trajectories over the illness course were traced using the CASH global severity ratings and functioning scales across the predefined time-periods (see Table 1). More specifically, we define the concept of symptom/syndrome load over the illness course as the highest severity of each psychopathological syndrome and its frequency of presentation within a given time-period. Similar to cross-sectional assessments, syndrome severity is scored from absent (0) to severe (5). Frequency of presentation is scored as 0 (never), 1 (at least once/rarely, <25% of the time), 2 (occasionally, about 25% of the time), 3 (often, about 50% of the time), 4 (very often, about 75% of the time), and 5 (always, 100% of the time). A lifetime score was calculated for each psychopathological domain by summing the severity and frequency scores of each period and dividing the score by the number of periods assessed. We also calculated a global psychopathology load score for each period by summing all six individual syndrome scores. The same rating scales to assess functioning at follow-up (see below) were also used to assess functioning trajectories over the illness course and the predefined periods.

#### Intervening Variables

Assessing intervening variables over the illness course is important, since the longer the time gap is between the background variables and the distal outcomes, the greater the possibility of intervening modifying effects. Following the methodology described above, information about drug abuse, medication history (type, mean dosage, duration), number of relapses, major life events, service use (i.e., number of hospitalizations) was also collated across the predefined course periods and over the entire illness course up to the follow-up assessment.

The following variables are assessed over the illness course (but not across the predefined periods): mental health service use other than admissions (i.e., number of emergency visits, number of visits at the mental health center), psychiatric and medical comorbidities, and total number of suicide attempts. Furthermore, lifetime medication use for antipsychotics, anticholinergics, antidepressants, mood stabilizers, and benzodiazepines was defined in terms of dose-years as rated per CASH, and the number of total sessions of Electro-Convulsive Therapy (ECT) is recorded. Using the same methodology to calculate dose-years of medication, we also estimate the dose-years of involvement in psychosocial rehabilitation programs and in psychotherapeutic programs conducted by a clinical psychologist. At follow-up, we also register current medication use such as involvement in psychological and psychosocial rehabilitation programs.

### Outcome Measures

We assess eight outcome domains at follow-up: psychopathology, psychosocial functioning, personal recovery, subjective quality of life, cognitive impairment, neuromotor dysfunction, comorbidity, and mortality rate (see Table 1).

#### Psychopathology

Psychopathology was primarily assessed with the current condition module from the CASH, which was used to rate symptoms over the last month, last 6 months, and last year in order to characterize symptom stability and persistence and to apply the RSWG criteria for symptom remission. Other measures of symptoms included the Bipolar Affective Disorder Dimension Scale (BADDS) (47) and the Schedule for the Deficit Syndrome (SDS) (48).

#### Psychosocial Functioning

Psychosocial functioning was rated as the best state of functioning over the last year by means of the GAF, the Social and Occupational Functioning Assessment Scale (SOFAS), the World Health Organization-Disability Assessment Schedule (WHO-DAS) (49), and the Functioning Assessment Short Test (FAST) (50). The areas of functioning covered by the WHO-DAS are personal care, familial, occupational, and social, which are rated on a 6-point Likert-type scale from 0 (impairment absent) to 5 (severe impairment). The areas of functioning covered by the FAST are autonomy, work, cognition, finance, relationships, and leisure, and they are rated on a 4-point scale ranging from 0 (impairment absent) to 3 (severe impairment). Combining the WHO-DAS and FAST, we evaluate seven different areas of functioning. Other functional outcomes that were assessed at follow-up were current civil status, paid employment, independent living, and modified legal capacity, all of which except the latter were also assessed at baseline. Previous studies have defined functional remission as sustained remission in functioning for ≥1 year or ≥2 years (17); thus, we defined functional remission according to the two criteria.

#### Personal Recovery

Self-rated personal recovery is a concept closely linked to quality of life that has gained support as a key outcome dimension. To assess it, we use the Questionnaire about the Process of Recovery (PRQ-15) (51), a standard measure of personal recovery developed in collaboration with service users (52). This instrument is a self-rated scale comprising 15 items covering the major components of personal recovery such as re-establishment of identity, finding meaning in life, taking responsibility for recovery, having sense of purpose, and hope (23). Subjects are asked to complete the questionnaire considering their state over the last year. A cut-off score ≥45 was used to define personal recovery from a categorical point of view.

#### Quality of Life

Self-perceived quality of life was assessed by means of the Schizophrenia Quality of Life Scale (SQLS) (53, 54), and the EUROQOL-5D-5L (55). The SQLS is a 30-item self-reported questionnaire measuring psychological, motivation, and energy dimensions, such as symptoms and side-effects. The EUROQOL-5D-5L is a self-rated instrument measuring five health dimensions (mobility, self-care, usual activities, pain/discomfort, and anxiety/depression) and a global rating for health status.

#### Neuromotor Dysfunction

Neuromotor dysfunction has been consistently linked to poor psychosocial and cognitive functioning and is deemed to stay close to the neurobiological underpinnings of the psychotic illness forming a specific phenomenological and outcome domain (56). Furthermore, it has been recently included as a specific field within the National Institute of Mental Health Research Domain Criteria (RDoC) framework (57). The Simpson-Angus rating scale (58) was used to assess Parkinsonism, the Barnes Akathisia Rating Scale (59) assessed akathisia, the Modified Rogers scale (60) rated a broad range of motor abnormalities, and the Neurological Assessment Scale (61) was used to rate neurological soft signs. Motor rates are made irrespective of their attribution as side-effects of medication, since, in subjects on antipsychotics, they are deemed to represent a mixture of primary and secondary phenomena (62, 63).

#### Cognitive Impairment

Cognitive performance was assessed by two clinical neuropsychologists (AST and GGB), who were blind to the clinical status of the subjects, by means of the Screen for Cognitive Impairment in Psychiatry (SCIP) (64, 65), the Montreal Cognitive Assessment (MoCA) (66), and the MATRICS Consensus Cognitive Battery (MCCB) (67). The SCIP was developed as a brief screening tool for the quantification of cognitive deficits that can be completed with a pencil and a timer and a total administration time of approximately 15 min. It contains five subtests quantifying working memory, immediate and delayed verbal list learning, verbal fluency, psychomotor speed, and an overall composite score. The MoCA includes assessments of the following functions: executive visuospatial, naming, memory, attention, language, abstraction, recall, and orientation. The MCCB has become the standard instrument for assessing cognitive impairment in psychotic disorders. It measures processing speed, attention/vigilance, working memory, verbal learning, visual learning, reasoning/problem solving, and social cognition.

#### Comorbidity

Comorbidity included the assessment of psychiatric and medical conditions. We evaluated the number and type of lifetime clinical diagnosis of mental disorders other than psychotic and major mood disorders. The presence of current metabolic syndrome is assessed by the criteria developed by the American Heart Association; National Heart, Lung, and Blood Institute (68) and its severity defined as the number of criteria present. Lastly, the number and type of current medical conditions other than the metabolic syndrome are rated such as the number and type of non-psychiatric medications prescribed.

#### Mortality

Mortality rate was assessed using electronic clinical records and official registers. We ascertained those patients who were dead during the follow-up period, and register the year of death and the definitive or probable cause of death, including suicide.

### Delineating Longitudinal Course Patterns and Staging

Course pattern trajectories of the subjects over the whole illness course were delineated using: (a) the DSM-5 descriptors of the course including full remission, partial remission, and continuous; (b) symptoms and functioning trajectories; and (c) the psychopathological and functional status at follow up. Types of course patterns and the psychopathological end state were classified according classical descriptions (69–71). For instance, illness course patterns were constructed using Ciompi's typology (71), which combines mode of onset (acute vs. insidious), overall symptom trajectory (episodic vs. continuous), and end state (recovered or minimal symptomatology vs. moderate or severe psychopathology).

Relatedly, we are specifically interested in describing the clinical staging of psychotic illness based on the long-term course, considering both classical descriptions of the illness course (72) and modern conceptualizations of staging (73). Using all available information on the entire illness course and a consensus approach among researchers involved in the study, we defined six stages. Stage 1: single episode with full remission; Stage 2: multiple episodes with full remission; Stage 3: multiple episodes with partial remission and stable levels of symptoms and/or functioning; Stage 4: multiple episodes with partial remission and progressing symptoms and/or declining functioning; Stage 5: chronic/continuous course with stable symptoms and functioning; and Stage 6: chronic/continuous course with progressing symptoms and/or declining functioning. A major advantage of this staging procedure is that it distinguishes elements of progression within the “partial remission” and “chronic/continuous stages,” as was emphasized in some classical studies (72) but obviated in modern studies. This clinical staging will be subjected to validation using the baseline and illness course variables.

### Data Analysis

Since the main study aim is the multivariate prediction of very long-term outcomes, we will follow the Transparent Reporting of a Multivariable Prediction Model for Individual Prognosis or Diagnosis (TRIPOD): the TRIPOD statement (74).

We first will examine the degree to which the follow-up sample was representative of the baseline sample regarding the main socio-demographics and clinical variables assessed at baseline. Second, we will descriptively examine the characteristics of the follow-up sample at several levels: antecedents; first-episode characteristics; longitudinal trajectories of psychopathology, and functioning including the criteria for symptomatic and functioning remission; and characteristics of the final outcomes. These descriptive analyses will entail important information about the characterization of the successive stages of the psychotic illness. We next will examine the degree to which outcome measures converge using Pearson's correlation coefficients for continuous variables and the kappa statistic for dichotomic variables.

To examine symptom and functioning trajectories over the longitudinal assessments, multivariate time-series analyses will be employed, and more specifically, vector auto-regression models will be applied. These models are particularly suited for investigating the temporal dynamics among two or more variables (75). We will also use Latent Class Growth Analysis (LCGA) for identifying distinct homogeneous subpopulations with similar symptom or functioning trajectories of growth over time (76). LCGA is a technique known as latent classes within longitudinal data collected from a larger heterogeneous population, and the estimated recovery trajectory classes will be compared on baseline predictor variables (77).

The predictive value of baseline variables for each outcome domain will be examined using hierarchical logistic or linear regression models. More specifically, Cox regression models will be used to account for time until follow-up and censoring of given categorical outcome. The intervening variables assessed over the illness course will be incorporated into the models using mediation analysis (78). Finally, we will determine the common and specific predictors of the different outcomes. To this end, we define “full clinical recovery” as symptomatic, functional, and personal recovery and will examine their predictors. To control for the potential confounding effects of age, sex, and years of follow-up, these variables will be added as covariates in multivariate analyses other than Cox regression.

From a network perspective, psychopathology is hypothesized to result from dynamic interactions among symptoms (79). Thus, we will use network analysis to examine whether the network structure of symptoms and their interactions at the first episode can predict illness course, outcomes, and network symptom structure at follow-up.

Given that it is expected that a majority of subjects at baseline change their diagnosis at follow-up, mainly to schizophrenia (80, 81), we will examine the antecedents and first-episode predictors of a diagnostic change by means of hierarchical logistic regression analysis. Furthermore, we will examine the prospective and retrospective stability of DSM-5 diagnoses of psychotic disorders.

## Discussion

Prediction models in medicine have proliferated in recent years and have been substantiated within the concepts of personalized and precision medicine. Moreover, health care providers and policy makers are increasingly recommending the use of prediction models within clinical practice guidelines to inform decision making at various stages of the clinical pathway. However, advances of knowledge are limited by the lack of implementation research in real-world clinical practice (82). Characterizing the long-term outcomes of psychotic disorders and identifying their baseline and clinical pathways represent crucial steps enabling risk-stratification and personalized, risk-adapted treatment. For instance, early identification of poor response to treatment would allow timely adjustments to management programs; additionally, as some predictors are modifiable, they may provide specific treatment targets.

Psychotic disorders are illnesses of unknown origin characterized by high variability in familial/genetic and environmental risk factors, heterogeneous clinical presentation, and a variety of outcomes; thus, the prediction of outcomes in psychotic disorders with reasonable clinical validity and utility continues to be a challenge. While there has been some consistent evidence about the predictive validity of some antecedent and first-episode variables on the symptomatic and functioning outcomes (26)—although with relatively poor predictive ability—much less is known about the predictors of other key outcome dimensions, such as the degree to which the different predictors influence the different outcomes.

The main hypothesis underlying this study protocol is that, given the heterogeneity of psychotic disorders at the level of risk factors, clinical presentation, and outcomes, the ability to predict the long-term outcomes of the psychotic illness can be substantially enhanced by considering a broad and comprehensive approach to the putative predictors, the mediating variables and a multidimensional concept of the outcome. We further hypothesize that the novel definitions of the lifetime psychopathological syndromes based on the concept of symptom load across the illness course would have greater validity than syndromes defined more cross-sectionally, and that the novel clinical staging based on the long-term illness course will have superior validity to the traditional characterizations of illness course and clinical staging.

This study protocol was built by broad and comprehensive approach to the putative baseline predictors, mediating variables, and a multidimensional concept of the outcome. Thus, the study has the potential of generating a predictive model of the long-term outcomes of psychotic disorders based on their baseline features, which can contribute to knowledge making us able in the future to better create such models applicable in clinical practice. Predictive models will improve the application of individually tailored, person-based interventions, adapted to one's current clinical condition, which will result in an enhanced overall treatment response, reducing the costs of mental disorders at personal, familial, and societal levels.

This study has a number of strengths. First, it was based on a well-characterized sample of subjects with FEP, who were assessed for a broad number of predictor variables at baseline including antecedents, first-episode characteristics, and early response to treatment. Second, multiple intervening variables influencing outcome are assessed over the illness course. Third, to the best of our knowledge, our study represents the most comprehensive approach to the outcome domains up to date, which will allow us to examine their common and specific determinants. Fourth, this study is one of the few with a very long-term follow-up design. Lastly, course and outcome information of the subjects was blindly collected regarding their baseline status.

The study results should be interpreted with some methodological considerations in mind. First, there might be a selection bias, since subjects who are willing to participate may differ from those who refuse. Second, the baseline sample consisted of subjects requiring hospitalization, which may reduce the generalizability of findings to less severely affected subjects. Third, the naturalistic design without a control group, including an age-matched healthy control group at follow-up, limits the possibility of examining causal relationships. Fourth, several of the outcome variables are only assessed at follow-up, and thus the study cannot investigate the development of these outcome measures. Fifth, the retrospective life chart assessment of longitudinal development conveys to possible recall bias, although the use of clinical records and key informants may to some extent compensate for this. Finally, health and social care unmet needs were not assessed, which limits the utility of our study in detecting areas of improvement in the health and care of the subjects.

## Ethics Statement

The studies involving human participants were reviewed and approved by Navarra Clinical Investigation Ethics Committee (CEIC project number 2016/71, 25th august 2017). The patients/participants provided their written informed consent to participate in this study.

## Author Contributions

VP and MC wrote the first draft and circulated it among the authors. LM-I, EG, AS-T, LJ, DP, and LF have carefully revised the manuscript and added some sections. All authors contributed to the article and approved the submitted version.

## Conflict of Interest

The authors declare that the research was conducted in the absence of any commercial or financial relationships that could be construed as a potential conflict of interest.

## SEGPEPSs Group

A. Ballesteros, G. Gil-Berrozpe, R. Hernández, R. Lorente, S. Papiol, M. Ribeiro, A. Rosero, R. Tabares-Seisdedos, and M. Zandio.

## Abbreviations

AHA: American Heart Association
AIMS: Abnormal Involuntary Movements Scale
ASS: Addition Severity Scale
BADDS: Bipolar Affective Disorder Dimension Scale
BARS: Barnes Akathisia Rating Scale
CASH: Comprehensive Assessment of Symptoms and History
CGI-EI: Clinical Global Impression Efficacy Index
C-SPAS: Cannon-Spoor Premorbid Adjustment Scale
DUP: Duration of Untreated Psychosis
DSM-III-R: Diagnostic and Statistical Manual of Mental Disorders 3rd edition, revised
DSM-IV: Diagnostic and Statistical Manual of Mental Disorders 4th edition
DSM 5: Diagnostic and Statistical Manual of Mental Disorders 5th edition
ECT: Electro-Convulsive Therapy
EUROQOL: European Quality of Life Group
FAST: Functioning Assessment Short Test
FEP: First episode psychosis
FLS: Familial Load Score
GAF: Global Assessment of Functioning
GFES: Global Family Environment Scale
IQ: Intelligence Quotient
LCGA: Latent Class Growth Analysis
LCS: Life Chart Schedule
L-M: Lewis-Murray Obstetric Complications Scale
MCCB: MATRICS Consensus Cognitive Battery
MoCA: Montreal Cognitive Assessment
MRS: Modified Rogers Scale
NDS: Neurodevelopmental Scale
NES: Neurological Evaluation Scale
QPR-15: Questionnaire about the Process of Recovery
PANSS: Positive and Negative Syndrome Scale
PRS: Polygenic Risk Score
RDoC: Research Domain Criteria
RSWG: Remission in Schizophrenia Working Group
SANS: Scale for the Assessment of Negative Symptoms
SAPS: Scale for the Assessment of Positive Symptoms
SARS: Simpson-Angus Rating Scale
SCIP: Screen for Cognitive Impairment in Psychiatry
SDS: Schedule for the Deficit Syndrome
SOFAS: Social and Occupational Functioning Assessment Scale
SQLS: Schizophrenia Quality of Life Scale
TRIPOD: Transparent Reporting of a Multivariable Prediction Model for Individual Prognosis or Diagnosis
WHO-DAS: World Health Organization-Disability Assessment Schedule
WHO-LCS: World Health Organization-Life Chart Schedule.
